# The ABC of tDCS: Effects of Anodal, Bilateral and Cathodal Montages of Transcranial Direct Current Stimulation in Patients with Stroke—A Pilot Study

**DOI:** 10.1155/2013/837595

**Published:** 2013-01-08

**Authors:** A. Fusco, D. De Angelis, G. Morone, L. Maglione, T. Paolucci, M. Bragoni, V. Venturiero

**Affiliations:** ^1^Clinical Laboratory of Experimental Neurorehabilitation, I.R.C.C.S. Santa Lucia Foundation, Via Ardeatina 306, 00179 Rome, Italy; ^2^Operative Unit F, I.R.C.C.S. Santa Lucia Foundation, Via Ardeatina 306, 00179 Rome, Italy; ^3^Physical Medicine and Rehabilitation, Sapienza University of Rome, Piazzale Aldo Moro 5, 00185 Rome, Italy

## Abstract

Transcranial direct current stimulation (tDCS) is a noninvasive technique that is emerging as a prospective therapy for different neurologic disorders. Previous studies have demonstrated that anodal and cathodal stimulation can improve motor performance in terms of dexterity and manual force. The objective of this study was to determine whether different electrodes' setups (anodal, cathodal, and simultaneous bilateral tDCS) provide different motor performance and which montage was more effective. As secondary outcome, we have asked to the patients about their satisfaction, and to determine if the bilateral tDCS was more uncomfortable than unilateral tDCS. Nine patients with stroke in subacute phase were enrolled in this study and randomly divided in three groups. Our results showed that tDCS was an effective treatment if compared to Sham stimulation (*P* = 0.022). In particular, anodal stimulation provided the higher improvement in terms of manual dexterity. Cathodal stimulation seemed to have a little effect in terms of force improvement, not observed with other setups. Bipolar stimulation seemed to be the less effective. No significant differences have been noted for the different set-ups for patients' judgment. These results highlight the potential efficacy of tDCS for patients with stroke in subacute phase.

## 1. Introduction

People with stroke results in several neurological impairments, affecting around 1 million subjects in Europe. Hence, stroke effects are the leading cause of long-term disability in industrialized societies [1, 2]. Rehabilitation's outcomes often conclude in incomplete motor recovery and over 60% of patients cannot use their paretic hands in functional activities. Furthermore, presence of severe paresis after four weeks is considered a negative predictive factor for the motor recovery [2], indicating for these patients serious difficulties in the activities of daily living in their future.

 To facilitate recovery of upper limb function, many different rehabilitative treatments are still proposing. Among them, researchers are focusing their attention on non-invasive brain stimulations (NIBS), worldwide. Tools of NIBS are the repetitive transcranial magnetic stimulation (rTMS) and the transcranial direct current stimulation (tDCS). 

Use of tDCS is increasing in patients with stroke for its modulatory effects on cognitive and motor functions [3–5]. In particular for the motor domain, the cortical target of tDCS application has been showed to enhance execution and skills [6], producing interest for the improvement of rehabilitative stroke's course. Moreover, respect than rTMS, it is less expensive, more mobile, and, therefore, more comfortable, making its use easier in clinical settings. 

This technique applies electrical current directly on the scalp and modulates the membrane potential dependently by type of electrode's application. In fact, anode is able to facilitate the depolarization of neurons, while, on the contrary, cathode hyperpolarizes the resting membrane potential, reducing the neuronal firing [7]. Application in motor domain for subjects with stroke has been showed to be effective in enhancing the performance of functional tasks and muscle force [4, 8, 9]. 

At the same time, a recent meta-analysis has underlined as small sample size, different setups, and a large effect size in studies concerning motor recovery on patients with stroke may reduce the clinical meanings of this preliminary evidence.

The aim of this study was to evaluate the effects on manual dexterity and pinch and grip force of a tDCS single stimulation, compared to Sham stimulation, and if this improvement was different among the three possible electrodes' montages (anodal, cathodal, or bipolar). Secondary outcome was to evaluate the satisfaction of patients in using this advanced rehabilitative technology. 

## 2. Material and Methods

This is a single-blind, crossover, sham-controlled study. Patients were admitted for an inpatient rehabilitation with a diagnosis of stroke to our hospital. The inclusion criteria to participate to this study were selected as follows: first-ever stroke; cortical or cortical-subcortical lesion, confirmed by diagnostic imaging (CT or MRI scans); mild to moderate hemiparesis with presence of minimal hand movement (proved by possibility to perform grip or pinch test). The following exclusion criteria were considered: presence of a history of chronic disabling pathologies of upper limb; spasticity; presence of pacemaker or severe cardiovascular conditions; a history of tumor, prior neurosurgical brain intervention, or severe cardiovascular conditions, including the presence of a pacemaker; a diagnosis of epilepsy or major psychiatric disorders. Demographic and clinical characteristics of the nine patients undergone the experimental procedure are summarized in Table 1.

The protocol was approved by the local independent ethics committee, and all participants gave written informed consent.

### 2.1. Transcranial Direct Current Stimulation

Stimulation was delivered for 15 minutes, both in real and sham condition, in two consecutive days, randomized for sham/tDCS and anodal/bipolar/cathodal stimulations. In both sessions, the stimulation was preceded by 60 seconds where the current was gradually increased until intensity of 1.5 mA, eliciting transient sensations that disappeared over seconds, consistently with previous reports [9, 10]. The stimulator (Eldith DC Stimulator, NeuroConn, Germany) provided the direct current using two gel-sponge electrodes with a surface area of 35 cm^2^ (5 × 7 cm for each electrode) embedded in a saline-soaked solution.

Positioning of active electrode varied according to randomized different montage: for anodal stimulation, the active electrode was placed on the projection of the hand knob area of the primary motor cortex of the affected hemisphere; for cathodal stimulation, the electrode was placed on unaffected hemisphere in an analogue position of the anodal stimulation. For these electrodes' setup, referent electrode was positioned on the skin overlying the contralateral supraorbital region. In bilateral montage, cathode and anode were positioned as active electrode in the same way described above. In the context of electrical stimulation, anode indicates the relative positive terminal where current flows into the body, while cathode indicates the relative negative terminal where the current exits from the body [11].

### 2.2. Test Protocol

Patients were asked to perform the 9-hole peg test (9HPT) before- and after-tDCS or Sham. This test consists of a squared board with 3 rows of 3 holes. Participants were asked to fill the 9-holes with pegs as fast as possible. Researchers recorded the time spent to execute the task with a stopwatch starting when the subject touched the first peg and stopping it when the subject filled the last hole or when time was longer than 50 s, as previous researches reported [12, 13]. 

Velocity of execution was computed in terms of holes filled per second (number of filled holes/time). 9HPT-index, as an index of manual dexterity, was obtained. To perform a data normalization among subjects, the 9HPT-index was computed as follows: 9HPT-index = velocity LS/velocity HS ∗ 100. The percentage improvement between pre and post treatment of 9HPT-index was computed as (9HPT-indexpost – 9HPT-indexpre)/9HPT-indexpre ∗ 100. 

As other outcome's measure, for each participant, the maximum pinch force and the maximum grasp force were measured by means of specific dynamometers. Both hands were evaluated with the patients seated, the elbow at 90° of flexion and a neutral position of the wrist. Grip force was determined according to Jamar method, with the arm as more stretched as possible and handlebars fixed to 5 cm, the most appropriate distance to develop the maximal force [14]. The maximum forces recorded between two trials were analyzed. Each participant performed these tests before- and after-tDCS or Sham. 

Finally, four questions were asked about the satisfaction with the tool from patients' perspective. Inspired by QUEST (Quebec User Evaluation of Satisfaction with Assistive Technology) questionnaire [15], items were concerned on dimension and utility of the device, modality of application, comfort in the using. Answers were graded on the Likert-type scale, the most widely used approach to scaling responses in survey research, from “not satisfied at all” to “very satisfied.”

### 2.3. Statistical Analysis

All measurements are reported in terms of mean ± standard deviation. A repeated measure analysis of variance was performed on the 9HPT-index using as within-subjects factors pre versus post treatment and tDCS versus Sham, whereas as between-subjects factor the type of setup (A, B, or C). Post hoc analyses had been performed using Tukey correction for the inflation or type I error for multiple comparisons. Similarly, same analyses were performed on the pinch and grasp forces recorded for the affected limb of subjects. For verifying the applicability of analysis of variance, we previously performed the Levene's test of equality of error variances for verifying the homogeneity of data of the 3 recorded variables (9HPT-index, pinch and grasp forces) for both the stimulations (tDCS versus Sham), before and after-stimulation. SPSS 17.0 was used and significant threshold was set at 0.05.

## 3. Results

Table 2 reports the experimental data of all nine selected patients.

Before applying analysis of variance, the data homogeneity was verified with Levene's test of equality of error variances, of the 3 recorded variables (9HPT-index, pinch and grasp forces) for both stimulations (tDCS versus Sham), before and after-stimulation. Eleven of 12 datasets resulted homogenous (*P* > 0.05), with a significant reduction of homogeneity observed just for grasping after sham stimulation (*P* = 0.039). According to these results, we applied repeated measure analysis of variance.

**Figure 1:**
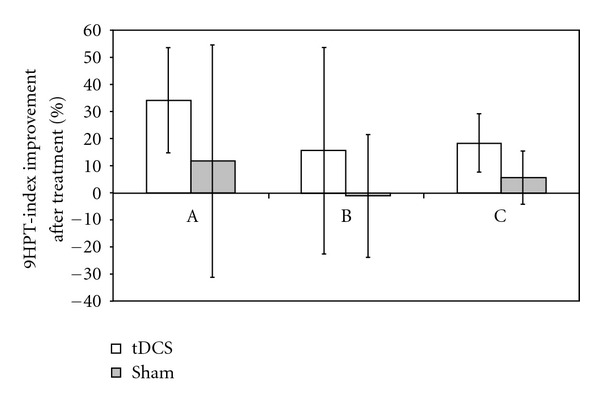
Percentual of improvement in the manual dexterity, measured as 9HPT index, for the real and sham stimulation in the three different electrodes' montages. Abbreviations for the stimulation: A: anodal; B: bilateral; C: cathodal.

The improvements recorded after tDCS treatment were significantly higher with respect to the changes observed after Sham treatment, as shown in Figure 1 and Table 3 (*P* = 0.022 of interaction Pre versus Post ∗ tDCS versus Sham). Despite the high data variability, anodal and cathodal showed the higher improvements, but the differences between setups were significant only as main factor (*P* = 0.008), but not for the interaction Pre versus Post ∗ tDCS versus Sham ∗ ABC (*P* = 0.212). Post hoc analyses revealed that the A group had a lower 9HPT-index already before treatment (*P* = 0.017, analysis of variance, factor group). 

In terms of manual force, the interaction among factors (Pre versus Post ∗ tDCS versus Sham ∗ ABC) significantly affected the pinch force of the affected limb (*F*(2,6) = 5.60, *P* = 0.042). Main factor ABC did not affect significantly the pinch force (*F*(2,6) = 1.22, *P* = 0.360). We found a significant improvement of +13.1 ± 7.5% after cathodal stimulation, a reduction of force of −6.5 ± 19.8% after bipolar stimulation and no changes (0% in mean) after anodic stimulation or Sham simulation. Grasping forces were not altered, with just a slight but not significant effect of tDCS versus Sham ∗ ABC interaction (*F*(2,6) = 4.00, *P* = 0.079), again with higher improvement after cathodal stimulation. 

Finally, regarding the user evaluation, overall satisfaction with the device was very good. Results of the short survey was reported in Table 4. 

**Figure 2:**
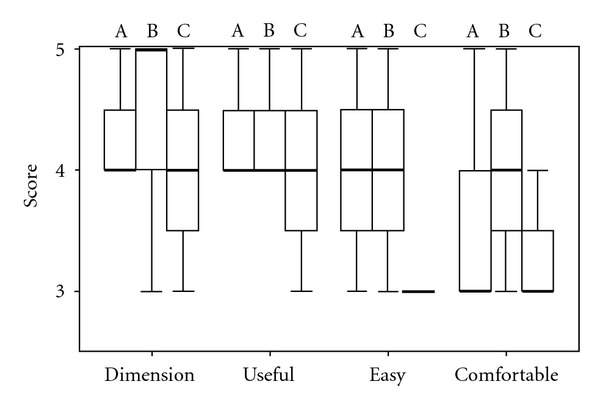
4-item user satisfaction questions, based on a Likert scoring, for the three electrodes' setups (A: anodic, B: bilateral, C: cathodic montage). Box (thin lines: first and third quartiles, wide line: median) and whiskers (minimum and maximum values) plot for patients' judgments about dimension, utility, easiness of use, and comfort.

No statistically significant changes were found among anodal, bilateral, and cathodal montages in terms of patients' judgment assessed by means of user satisfaction scale in terms of dimension (*P* = 0.848, Kruskal-Wallis analysis), perceived utility (*P* = 0.846), perceived easiness to use (*P* = 0.230), and comfort during treatment (*P* = 0.656) (Figure 2). Just a little surprising trend was observed indicating that bipolar montage was perceived as less invasive, despite the presence of two electrodes on the head and being more comfortable.

## 4. Discussion

The purpose of this study was to determine the effects of a single transcranial direct current stimulation (real versus sham) on dexterity and manual force in patients with stroke, performing the stimulation through three different electrodes' setups, and determining if the montage was perceived by the patients as satisfactory. Our results suggest that tDCS treatment was more effective than Sham treatment on manual dexterity, while no significant differences were recorded in terms of manual force, even if a slight improvement was noted after the cathodal stimulation. Furthermore, no difficulties in performing treatment were complained by the patients.

Recently, many studies have been focused on devices to facilitate the motor recovery. The tDCS is emerging as one of the most interesting device to apply in stroke rehabilitation, both for the cognitive and motor impairments. Treatments with tDCS can be supplied up to 30 minutes, similarly to the timing of rehabilitative session, before or in synchrony with it, enhancing the rehabilitation outcomes [9, 10]. Moreover, compared to other forms of NIBS, tDCS is more comfortable, more mobile, and cheaper, and no major adverse effects have been reported. Common side effects include mild headache, itching, and erythema at the electrode site [16]. 

In spite of these advantages, use of this technique in rehabilitation is counteracted due to still too preliminary evidence. In fact, studies vary widely in terms of phase of stroke, functional impairments, targeting of the outcomes, stimulation set-ups, and rehabilitative integration. Hence, in a recent meta-analysis, Bastani and Jaberzadeh concluded that tDCS (in that case, as anodal stimulation) seems to produce significant effects in subjects with stroke but any conclusion should be considered cautiously [3]. At the same, they also noted its potential role as add-on technique to improve motor function and corticomotor excitability.

In our study, we have focused our attention on different electrodes' montages, being an increasing interest on type of stimulation. Our results show as anodal stimulation provided the higher improvement in terms of manual dexterity. These findings are consistent with previous reports [8, 17–19]. In such cases, effects can last up to 2 weeks after the treatment [17]. Most of these studies are concerning a chronic phase of the stroke, while only Kim and colleagues have showed a stimulation effect on patients in a subacute phase. Noteworthy, a recent report has observed that tDCS does not seem to be effective in an acute phase [20]. 

The tasks utilized to measure the manual dexterity, including the Jebsen-Taylor test, the Box and Block test, and the 9HPT, need a complex sensory information and sensorimotor integration for accurate performance. Moreover, the successful performance requires a complex pattern of activation of muscles and joints as well as the use of targets and tools [9, 21]; hence, the role of enhancer of motor rehabilitation should be more appropriate for the anodal stimulation of tDCS.

In fact, also the stimulation with cathode of the unaffected hemisphere seems to be effective in motor function improvement, but reports are not always concordant [17, 19]. On the contrary, our results showed that cathodal tDCS seemed to be a little effect in terms of force, differently by other setups.

In our study, bipolar stimulation seemed to be the less effective. In a previous study, it was reported as simultaneous application of anodal tDCS over the motor cortex and cathodal tDCS over the contralateral motor cortex induced an increase in cortical excitability [22]. Our study supports these findings in terms of dexterity, suggesting a global effect of treatments based on electrical stimulation respect than sham conditions, also for the bipolar montage of electrodes.

Finally, the overall satisfaction by the patient was maintained during a brief protocol treatment, confirming the facility of using this device [10].

The main limitation of our study was the reduced sample size. Although the number of subjects involved in this study was in line with other studies on tDCS [4, 8–10, 17–19], it suggests cautions in data interpretation. On the other hand, from a statistical point of view, the significant effects found in our study (*P* = 0.022 for interaction Pre versus Post ∗ tDCS versus Sham for 9HPT-index and *P* = 0.042 for interaction Pre versus Post ∗ tDCS versus Sham ∗ ABC for pinch force) obtained on a small sample were potentially larger than equivalent results obtained with larger samples, supporting the importance of our results. Anyway, further researches on wider samples are needed. Moreover, the group of anodal stimulation had a generally lower manual dexterity (but not force) that could limit the interpretation of our results. Further research on larger samples is hence needed.

In conclusion, the present study contributes to the panel of evidence that strengthen the role of tDCS inside the rehabilitative stroke's course, in particular for the more complex activities of daily living as add-on technique. More studies are needed to define the better montage set-ups, targeting more specific outcome measures.

## Figures and Tables

**Table 1:** Demographic and clinical characteristics of participants.

	Patient (number-initial of surname)									
	1-A.	2-R.	3-A.	4-S.	5-B.	6-D.M.	7-O.	8-R.	9-V.	Mean & S.D.
Age	36	27	57	67	82	33	65	37	78	53.5 ± 20.7
Gender (male/female)	F	F	M	M	M	F	M	F	M	
Handedness (right/left)	R	R	R	R	R	R	R	R	R	
Type of lesion (hemorrhagic/ischaemic)	I	I	I	I	I	I	I	I	H	
Time after stroke (days)	29	36	47	21	32	22	32	10	26	28.3 ± 10.4
Site of hemiparesis (right/left)	R	R	L	R	L	L	R	L	R	
Type of tDCS (anodal/bipolar/cathodal)	B	A	A	A	B	C	B	C	C	
Sequence of stimulation (tDCS/Sham)	T-S	T-S	T-S	S-T	S-T	S-T	T-S	T-S	S-T	

Mean ± standard deviation of demographic characteristics and clinical features are reported. Abbreviations in the table above: M: male; F: female; R: right; L: left; H: hemorrhagic stroke; I: ischaemic stroke; A: Anodal; B: Bipolar; C: Cathodal; T: tDCS stimulation; S: sham stimulation; S.D.: standard deviation.

**Table 2:** Data of 9HPT-index and manual force recorded for each patient.

Type of stimulation	Type of setup stimulation	Prestimulation			Poststimulation		
		9HPT-index (%)	Pinch (kg)	Grasp (kg)	9HPT-index (%)	Pinch (kg)	Grasp (kg)
tDCS	Anodal	26.7	3.5	14	28.0	3.5	12
tDCS	Anodal	25.6	3.5	10	29.6	3.5	9
tDCS	Anodal	19.6	6	18	24.0	6	18
tDCS	Cathodal	44.7	4.5	14	48.3	6	12
tDCS	Cathodal	88.9	5.5	16	75.0	6	22
tDCS	Cathodal	81.1	2.5	14	100.0	4.5	14
tDCS	Bilateral	88.9	6	24	75.0	5	15
tDCS	Bilateral	77.3	9.5	34	88.9	11	36
tDCS	Bilateral	57.1	5	18	75.0	2.5	16
Sham	Anodal	35.1	4	10	21.3	4	10
Sham	Anodal	20.4	2	8	26.7	2	10
Sham	Anodal	22.2	5	14	25.3	5	16
Sham	Cathodal	30.2	3	15	32.0	3	14
Sham	Cathodal	70.6	6	22	61.1	6	20
Sham	Cathodal	106.3	4	18	96.6	4	20
Sham	Bilateral	125.0	6	16	94.4	6	18
Sham	Bilateral	93.3	9	36	77.8	10	38
Sham	Bilateral	133.3	4.5	20	125.0	3.5	20

**Table 3:** Repeated measure ANOVA results.

Factors and interactions	df	*F*	*P*
Pre versus Post	1	0.475	0.516
Pre versus Post ∗ ABC	2	0.404	0.685
tDCS versus Sham	1	1.457	0.273
tDCS versus Sham ∗ ABC	2	3.167	0.115
Pre versus Post ∗ tDCS versus Sham	1	**9.507**	**0.022**
Pre versus Post ∗ tDCS versus Sham ∗ ABC	2	2.030	0.212
ABC	2	**11.808**	**0.008**

df: degrees of freedom (df of error = 6), *F* and *P* values (in bold if statistically significant).

**Table 4:** 4-item satisfaction of the user and the 5-point scale used to rate each item.

	Patient (number-initial of surname)									
	1-A.	2-R.	3-A.	4-S.	5-B.	6-D.M.	7-O.	8-R.	9-V.	Mean & S.D.
Dimension	5	5	4	4	3	5	5	4	3	4.2 ± 0.8
Utility	4	5	4	4	4	5	5	4	3	4.2 ± 0.6
Application	4	5	3	4	3	3	5	3	3	3.6 ± 0.9
Comfort	4	5	3	3	3	4	5	3	3	3.6 ± 0.9

1: not satisfied at all, 2: not very satisfied, 3: more or less satisfied, 4: quite satisfied, and 5: very satisfied. Abbreviations in the table above: S.D.: standard deviation.
